# Effects of COPD on Left Ventricular and Left Atrial Deformation in Patients with Acute Myocardial Infarction: Strain Analysis Using Speckle-Tracking Echocardiography

**DOI:** 10.3390/jcm11071917

**Published:** 2022-03-30

**Authors:** Julian Grebe, Tobias Müller, Ertunc Altiok, Michael Becker, András P. Keszei, Nikolaus Marx, Michael Dreher, Ayham Daher

**Affiliations:** 1 Department of Cardiology, Angiology and Intensive Care Medicine, University Hospital RWTH, 52074 Aachen, Germany; jgrebe@ukaachen.de (J.G.); ealtiok@ukaachen.de (E.A.); michael.becker@rheinmaasklinikum.de (M.B.); nmarx@ukaachen.de (N.M.); 2 Department of Pneumology and Intensive Care Medicine, University Hospital RWTH, 52074 Aachen, Germany; tobmueller@ukaachen.de (T.M.); mdreher@ukaachen.de (M.D.); 3 Department of Cardiology, Nephrology and Intensive Care Medicine, Rhein-Maas Hospital, 52146 Wuerselen, Germany; 4 Center for Translational & Clinical Research Aachen (CTC-A), University Hospital RWTH, 52074 Aachen, Germany; akeszei@ukaachen.de

**Keywords:** COPD, echocardiography, left atrium, left ventricle, global longitudinal strain, myocardial dysfunction

## Abstract

Myocardial strain analysis, which describes myocardial deformation (shortening or lengthening), provides more detailed information about left ventricular (LV) and atrial (LA) functions than conventional echocardiography and delivers prognostic information. To analyze the effects of COPD on left heart function upon acute myocardial infarction (AMI), consecutive AMI patients were retrospectively screened, and patients were included if a post-AMI echocardiography and results of recent pulmonary function tests (PFTs) were available. Strain analysis was performed by a cardiologist who was blinded to clinical information. Overall, 109 AMI patients were included (STEMI: 38%, non-STEMI: 62%). COPD patients (41%) had significantly more impaired LV “global-longitudinal-strain” (LV-GLS) compared to non-COPD patients (−15 ± 4% vs. −18 ± 4%; *p* < 0.001, respectively), even after adjusting for LV-ejection-fraction (LVEF) and age (mean estimated difference: 1.7%, *p* = 0.009). Furthermore, COPD patients had more impaired LA strain (LAS) than non-COPD patients in all cardiac cycle phases (estimated mean differences after adjusting for LVEF and age: during reservoir phase: −7.5% (*p* < 0.001); conduit phase: 5.5% (*p* < 0.001); contraction phase: 1.9% (*p* = 0.034)). There were no correlations between PFT variables and strain values. In conclusion, the presence of COPD was associated with more impaired LV and LA functions after AMI, as detected by strain analysis, which was independent of age, LVEF, and PFT variables.

## 1. Introduction

Chronic obstructive pulmonary disease (COPD) and coronary artery disease (CAD) represent major causes of mortality worldwide, and the combination of both diseases is particularly unfavorable in terms of prognosis [1,2]. Besides that, a growing body of evidence shows that COPD is not only a lung disease with pathological abnormalities in airways and lung parenchyma, but it is rather a systemic disease with a major impact on occurrence and prognosis of CAD [2,3], and on outcomes after acute myocardial infarction (AMI), affecting success and complications of revascularization therapy, development of heart failure, and mortality rates [4,5,6,7,8,9]. However, while echocardiographic assessment of left ventricular ejection fraction (LVEF) after AMI has an established role in risk stratification [10], echocardiographic studies assessing LVEF in COPD patients after AMI are very heterogeneous and not conclusive [11,12]. Nevertheless, several studies showed that assessing left ventricular (LV) function using additional modalities like global longitudinal strain (GLS) analysis, which describes the cyclic deformation (i.e., shortening or lengthening) of the myocardium, gives better information about LV dysfunction than left ventricular ejection fraction (LVEF), and importantly provides additional prognostic information [13]. In addition, data from retrospective studies on patients with ST-segment elevation myocardial infarction (STEMI) demonstrated that COPD patients exhibit a more impaired LV-GLS than patients without COPD, suggesting a greater functional impairment early after STEMI, which correlates with the development of heart failure and an increased all-cause mortality in COPD patients [12,14]. However, pulmonary function tests (PFTs) were not routinely performed to diagnose and quantify the severity of COPD in these studies. This is likely to be an important limitation because approximately 80% of people with COPD are undiagnosed [15], and 20–50% of those with self-reported COPD do not fulfill spirometric disease criteria [16]. Furthermore, patients with non-STEMI (NSTEMI) were not included in these studies, which also represents a significant limitation since COPD patients more often present with NSTEMI instead of STEMI as compared to non-COPD patients [17]. Besides, none of these studies analyzed the left atrial strain (LAS) using speckle-tracking echocardiography, though LAS represents significant prognostic information after AMI [18]. Therefore, the aim of our study was to assess abnormalities in LV and left atrial (LA) function after AMI (STEMI and NSTEMI) in patients with and without COPD using speckle-tracking echocardiography, and to analyze the relationships of LV and LA strain values with PFT variables.

## 2. Materials and Methods

The protocol for this retrospective study was approved by the local ethics committee (The Independent Ethics Committee at the RWTH Aachen Faculty of Medicine, EK 041/21), and the study was performed in accordance with the ethical standards laid down in the Declaration of Helsinki in its latest revision. Due to the retrospective study design, the requirement for informed consent to participate has been waived by the local ethics committee.

**Figure 1:**
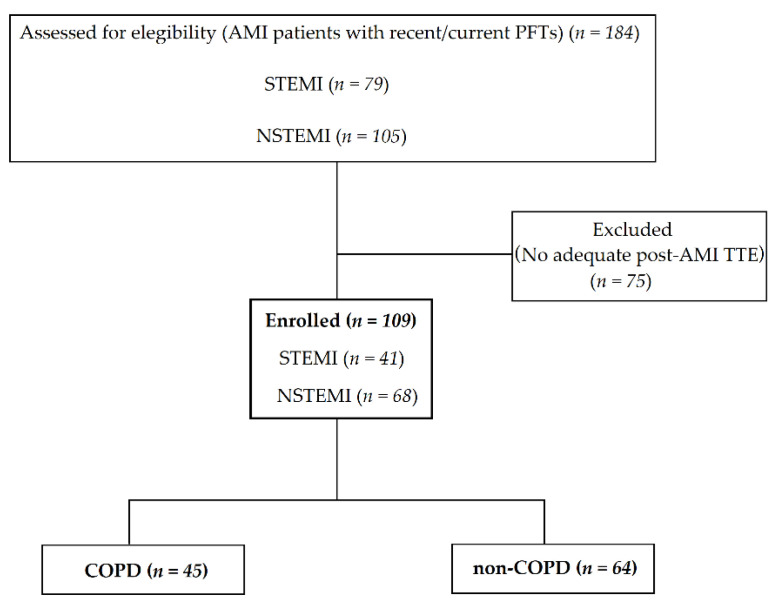
Consort flow chart showing how the analytical sample was derived from the patients who were assessed for eligibility. Abbreviations: AMI, acute myocardial infarction; COPD, chronic obstructive pulmonary disease; NSTEMI, non ST-segment elevation myocardial infarction; PFTs, pulmonary function tests; STEMI, ST-segment elevation myocardial infarction; TTE, transthoracic echocardiogram.

All patients admitted to our institution due to AMI between January 2015 and December 2019 were retrospectively screened for eligibility (Figure 1).

Patients were only included if results of current or recent (not older than six months prior to AMI) pulmonary function test (PFTs) and a post-AMI transthoracic echocardiography with an adequate image quality were available. Demographic data, disease history, coexisting medical conditions, smoking, and medication history were retrieved for all patients. Patients’ data were retrieved from the patient data management system (CGM MEDICO; CompuGroup Medical Clinical Europe GmbH, Koblenz, Germany).

Echocardiography was performed by primary investigators with commercially available ultrasound systems (Vivid E9 with a M5S probe and Vivid E95 with a 4Vc probe, GE Vingmed Ultrasound, Horten, Norway). Standardized echocardiographic measurements were obtained by primary investigators in accordance with the guidelines of the EACVI (European Association of Cardiovascular Imaging) and ASE (American Society of Echocardiography). LVEF was measured in 4 chamber and 2 chamber views by Simpson’s method of discs.

Whole-body plethysmography (MasterLab; Viasys, Hoechberg, Germany) was performed by primary investigators before and after bronchodilation according to current guidelines and recommendations [19,20,21]. Samples for arterial blood gases (ABG) analyses were taken from the arterialized earlobes of all patients while breathing room air without supplemental oxygen (ABL 800 flex; Radiometer, Copenhagen, Denmark).

**Figure 2:**
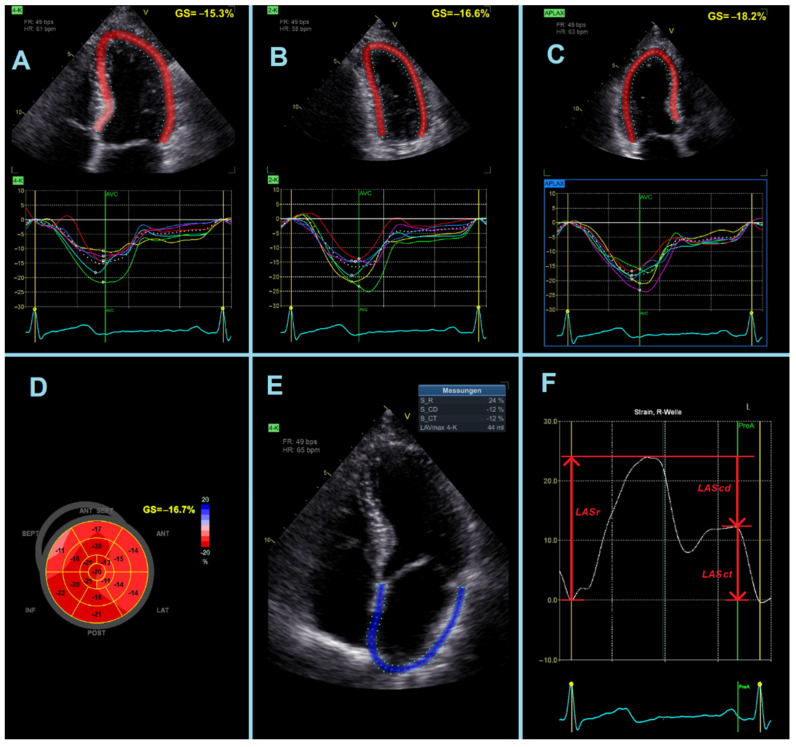
Speckle tracking echocardiography with left ventricular global longitudinal strain (LV-GLS) assessment (**A**) apical 4-chamber view; (**B**) apical 2-chamber view; (**C**) apical 3-chamber view; (**D**) Bull’s-eye plot), and left atrial strain assessment (LAS) in apical 4-chamber view (**E**,**F**).

At the time of analysis, patients were stratified into the two following subgroups: the presence of COPD versus no COPD, according to the GOLD criteria [22]. Strain analysis was performed using speckle-tracking echocardiography by a cardiologist with more than 6 years of experience in echocardiographic analysis, who was blinded to all clinical information, including the type of AMI, PFTs, and presence of COPD. Data were analyzed off-line using a customized software package (EchoPAC Version 204, GE Vingmed Ultrasound, Horten, Norway). Strain analysis by speckle-tracking echocardiography was performed when the examiner judged the image quality to be adequate with good tissue tracking. LV-GLS was measured using standard 2D grayscale images of the LV, which were acquired from conventional apical 4-, 2- and 3-chamber views according to the recommendations of the EACVI and ASE (Figure 2A–D) [23].

LAS values were measured as recommended by the “EACVI/ASE/Industry Task Force” in apical 4-and 2-chamber views and were reported separately for the three phases of LA cycle: reservoir, conduit, and contraction phase (Figure 2E,F) [24]:

- LASr = strain during reservoir phase, measured as the strain value from the ventricular end-diastole to the mitral valve opening at ventricular end-systole (positive value).
- LAScd = strain during conduit phase, measured as the strain value from the mitral valve opening to the onset of atrial contraction (negative value). In patients with atrial fibrillation, LAScd has the same value as LASr, but with a negative sign.
- LASct = strain during contraction phase, measured only in patients in sinus rhythm as the strain value from the onset of atrial contraction to ventricular end-diastole (negative value).

Data were summarized using absolute frequencies, percentages, means, standard deviations, and quartiles within the study sample and for patients with and without COPD. Data entry errors were identified with the help of plotting each variable and identifying outliers. Groups were compared using the Kruskal–Wallis rank sum test and Pearson chi-squared test. Pearson correlation coefficients were calculated to measure the strength of linear associations. Differences in mean strain values between COPD/non-COPD groups were estimated using linear models (linear regression analysis) with and without adjustment for LVEF, age, and when measurable for maximum tricuspid regurgitation velocity, which was measured as a surrogate for pulmonary artery pressure. Along with the parameter estimates, 95% confidence intervals were calculated. The R software for statistical computing was used for calculations (R Core Team (2018). R: A language and environment for statistical computing. R Foundation for Statistical Computing, Vienna, Austria. URL https://www.R-project.org/ (accessed on 9 February 2022); version 3.5.2).

## 3. Results

A total of 184 AMI patients who had results of current/recent PFTs were screened for eligibility (Figure 1), of whom 109 patients (59%) had post-AMI echocardiography with adequate image quality. Of the 109 patients who had met the inclusion criteria (age 65 ± 12 years, 78% male), 45 patients (41%) had COPD (forced expiratory volume in 1 s/forced vital capacity (FEV1/FVC): 58 ± 9%) and 64 (59%) had no COPD (FEV1/FVC: 80 ± 5%). Overall, 41 patients (38%) had STEMI at admission and 68 patients (62%) had NSTEMI. COPD patients were older than those without COPD (68 ± 11 vs. 63 ± 12 years, *p* = 0.05, respectively). Regarding the type of AMI (STEMI vs. NSTEMI), an 11 percentage point difference between patients with and without COPD was not statistically significant (STEMI 31% and 42% in patients with and without COPD, respectively; Chi-squared: 1.38; d.f:1, *p* = 0.24). Furthermore, patients with and without COPD did not differ in terms of additional co-morbidities, including atrial fibrillation, whereas—as expected—there were significant differences with respect to PFT variables. Additional demographic data, co-morbidities, and medications at admission are described in Table 1.

**Figure 3:**
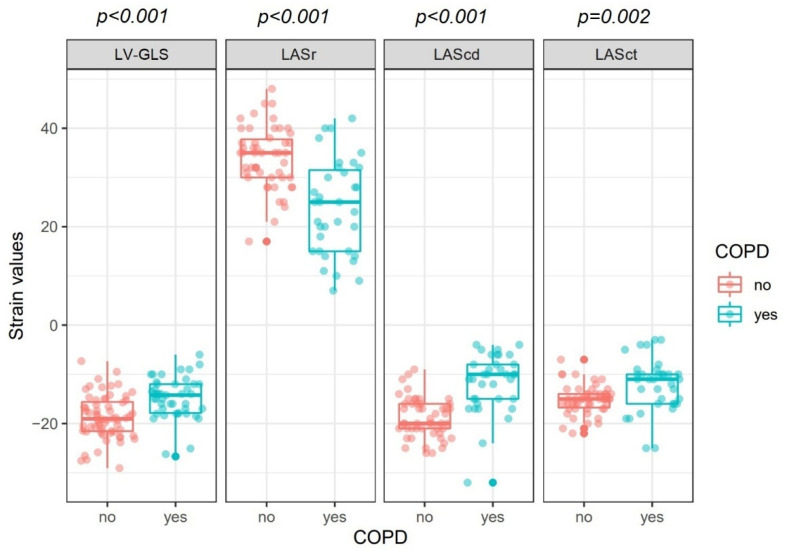
Left ventricular and left atrial strain values among patients with and without COPD. Abbreviations: COPD, chronic obstructive pulmonary disease; LAScd, left atrial strain during conduit phase; LASct, left atrial strain during contraction phase; LASr, left atrial strain during reservoir phase; LV-GLS, left ventricular global longitudinal strain.

Concerning echocardiographic variables, LVEF was lower in COPD patients (48 ± 9 vs. 53 ± 7%, *p* = 0.01,). Furthermore, COPD patients had significantly more impaired LV-GLS values compared to patients without COPD (−15 ± 4% vs. −18 ± 4%; *p* < 0.001), (estimated differences in means between patients with and without COPD for LV-GLS 3.8% (standard error (SE) = 0.9), *p* < 0.001) (Table 2 and Figure 3).

**Figure 4:**
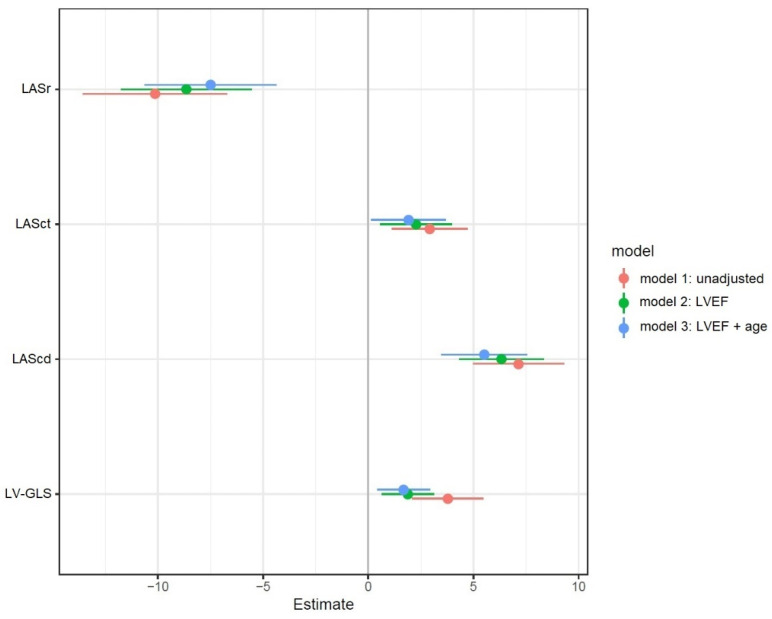
Estimated differences in strain values between patients with and without COPD (strain value of COPD patients—strain value of non-COPD patients). Positive estimates indicate larger (more positive or less negative) values among COPD patients. Model 1: unadjusted; model 2: adjusted for LVEF (%); model 3: adjusted for LVEF (%) and age (years). Abbreviations: COPD, chronic obstructive pulmonary disease; LAScd, left atrial strain during conduit phase; LASct, left atrial strain during contraction phase; LASr, left atrial strain during reservoir phase; LVEF, left ventricular ejection fraction; LV-GLS, left ventricular global longitudinal strain.

The difference in LV-GLS between patients with and without COPD remained significant even after adjusting for LVEF (estimated differences in means for LV-GLS: 1.8%, SE = 0.6; *p* = 0.003), after adjusting for LVEF and age (estimated differences in means for LV-GLS: 1.7%, SE = 0.6; *p* = 0.009) (Figure 4) and after adjusting for maximum tricuspid regurgitation velocity (Supplementary Figure S1). Among patients with COPD, the presence of COPD medications at admission showed no significant correlation with strain values after AMI (Supplementary Figure S2).

Left atrial size was more enlarged in COPD patients (LA-volume: 61 ± 29 mL vs. 49 ± 17 mL, *p* = 0.03; left atrial volume index (LAVI): 32 ± 14 vs. 25 ± 9 mL/m^2^, *p* = 0.01; respectively) (Table 2), along with more impaired LAS in all cardiac cycle phases (estimated differences in means between patients with and without COPD: for LASr −10% (SE: 1.7; *p* < 0.001); LAScd 7% (SE: 1.1; *p* < 0.001); LASct 3% (SE: 0.9; *p* = 0.002)) (Figure 3).

The difference in LAS between patients with and without COPD remained significant even after adjusting for LVEF and age (Figure 4): estimated differences in means between patients with and without COPD after adjusting for LVEF: LASr −9% (SE: 1.6; *p* < 0.001), LAScd 6% (SE:1.0; *p* < 0.001), LASct 2% (SE: 0.9; *p* = 0.01); estimated differences in means between patients with and without COPD after adjusting for LVEF and age: LASr −7.5% (SE: 1.6; *p* < 0.001), LAScd 5.5% (SE: 1.0; *p* < 0.001), LASct 1.9% (SE: 0.9; *p* = 0.034) (Figure 4).

By analyzing the relationship between PFT variables and strain values, there were no significant correlations.

## 4. Discussion

The present study showed that COPD is associated with more impaired left ventricular and left atrial deformation after AMI as measured by strain analysis. These abnormalities could be missed when LV function is only quantified by LVEF, as the differences in strain values between both groups were significantly different even after adjustment for LVEF and age. Conversely, pulmonary function test variables did not correlate with strain values in either group (COPD and non-COPD). It is possible that COPD-related burdens other than mechanical effects could influence left cardiac function after AMI.

The presence of COPD is known to worsen short- and long-term outcomes as well as prognosis after AMI [2,25]. Outcomes like recurrent AMI, need for coronary revascularization, development of heart failure, cardiac arrhythmias, and most importantly, long-term post-discharge cardiac death after AMI occur more frequently in COPD patients irrespective of LVEF [2,4,5,25,26,27]. This relationship is still not fully understood, and while some evidence suggests that COPD patients tend to have larger infarct sizes [11,28,29], other studies demonstrated that COPD basically increases cardiomyocyte stress and fibrosis [30]. Although the exact pathophysiologic mechanisms are still largely unexplained, the need for adequate risk stratification to identify patients at risk for adverse events has been recognized as an unmet clinical need [25]. However, whilst data about LVEF assessment after AMI in COPD patients is heterogeneous, LV-GLS has been identified as a prognostic marker in various cardiac diseases [25]. In our study, LV-GLS values were more impaired in patients suffering from COPD; and the difference in LV-GLS between patients with and without COPD was still significant after adjusting for LVEF and for both LVEF and age, respectively, suggesting a subtle left ventricular dysfunction after AMI in COPD patients which might be missed when only conventional measures for LV function are used. Furthermore, detecting LV dysfunction via LV-GLS might have significant clinical implications since LV-GLS is known to correlate with long-term prognosis [13]. In accordance, LA dysfunction measured by left atrial strain was also more pronounced in COPD patients after AMI, independently of LVEF and age. Of note, left atrial dysfunction has been shown to be of additional predictive value after AMI and is associated with all-cause mortality [18].

Changes in intrathoracic volume and pressure could serve as a potential explanation for the association between COPD and left heart dysfunction after AMI. However, in our study, there were no correlations between PFT variables and LV and LA strain values. Neither dynamic nor static lung volumes correlated with strain values, and parameters of hyperinflation also showed no correlations with LV or LA strain values. Overall, the pathophysiologic mechanisms of COPD-related myocardial dysfunction are still unclear and it is likely that multiple mechanisms are involved, including cardiomyocyte dysfunction due to low-grade inflammation or oxidative stress as well as enhanced LV remodeling and fibrosis [25,31,32], which may be detected using LV-GLS [25]. Furthermore, COPD patients were shown to have sub-clinical LV dysfunction even in patients with mild airway obstruction, suggesting that cardiac co-morbidities commence early in the development of COPD [33]. The presence of this subclinical myocardial dysfunction detected by LV-GLS was shown to independently predict all-cause mortality in COPD patients even among subjects with normal LVEF [34]. Furthermore, cardiac magnetic resonance studies have shown that COPD patients do have an expansion of the myocardial extracellular volume suggestive of diffuse myocardial fibrosis associated with LV remodeling [35]. Regarding the left atrium, the impairments of LAS in our study were also independent of PFT variables, so the assumed mechanical explanation for this relationship is not sufficient. COPD-related chronic systemic inflammatory status might also lead to atrial remodeling, resulting in decreased atrial function [36].

One limitation of our study is the lack of follow-up data. This would be very interesting, and future studies are needed to define risk groups based on cardiac strain analysis in COPD patients after AMI. This might help to optimize the treatment of both CAD and COPD. Another limitation is that 41% of patients were excluded because echocardiograms were not available after AMI or because the echocardiographic image quality was inadequate. However, previous studies have reported that up to 28% of echocardiograms are not suitable for LV-GLS analysis [37]. The higher proportion of excluded patients in our study is explained by some missing post-AMI echocardiograms but also by the unusual characteristics of this cohort with limited acoustic windows in many patients.

## 5. Conclusions

COPD is associated with more impaired left ventricular and left atrial functions after AMI, as detected by LV global longitudinal strain and LA strain analyses. These abnormalities could not be explained by impaired lung volumes and might be due to other pathophysiologic mechanisms of the systemic disease COPD.

## Figures and Tables

**Table 1:** Patients’ clinical and demographic data.

	All Patients (*n* = 109)	COPD (*n* = 45)	Non-COPD (*n* = 64)	*p*-Value
Age, years	65 ± 12	68 ± 11	63 ± 12	0.05
Male	85 (78%)	34 (76%)	51 (80%)	0.61
Female	24 (22%)	11 (24%)	13 (20%)	0.61
Body-Mass-Index, kg/m^2^	27 ± 5	27 ± 5	27 ± 4	0.67
NSTEMI	68 (62%)	31 (69%)	37 (58%)	0.24
STEMI	41 (38%)	14 (31%)	27 (42%)	0.24
Laboratory tests				
- Serum creatinine, mg/dL	0.99 ± 0.34	1.05 ± 0.45	0.95 ± 0.23	0.56
- GFR, mL/min/1.73m^2^	78 ± 21	73 ± 23	82 ± 19	0.06
- NT-proBNP, pg/mL	1273 (425; 2344)	1379 (422; 2323)	1238 (449; 2316)	0.74
- CRP, mg/dL	9 (3; 37)	5 (2; 29)	13 (5; 42)	0.14
- CK_max_, U/L	535 (226; 1385)	395 (133; 1197)	607 (259; 1478)	0.32
- cTnT_max_, pg/mL	767 (169; 2390)	365 (102; 1632)	1125 (210; 2784)	0.17
Smoking history				
- Current smoker	49 (45%)	23 (51%)	26 (41%)	0.07
- Ex-smoker	43 (39%)	22 (49%)	21 (33%)	0.07
- Never smoked	17 (27%)	0 (0%)	17 (27%)	0.07
- Period of smoking, years	33 ± 14	35 ± 13	31 ± 15	0.45
- Smoking, Pack-year	37 ± 29	45 ± 29	33 ± 28	0.03
Co-morbidities				
- Hypertension	81 (74%)	34 (76%)	47 (73%)	0.80
- Diabetes	28 (26%)	14 (31%)	14 (22%)	0.28
- Bronchial asthma	5 (5%)	3 (7%)	2 (3%)	0.38
- Atrial fibrillation	27 (25%)	10 (22%)	8 (13%)	0.18
Medications at admission				
- ACE inhibitor/AT_1_ antagonist	69 (63%)	29 (64%)	40 (63%)	0.99
- Aspirin	64 (59%)	30 (67%)	34 (53%)	0.22
- Beta blocker	58 (53%)	21 (47%)	37 (58%)	0.34
- Statin	58 (53%)	28 (62%)	30 (47%)	0.17
- P_2_Y_12_ inhibitor	19 (17%)	10 (22%)	9 (14%)	0.40
- CCB	17 (16%)	10 (22%)	7 (11%)	0.18
- Loop diuretic	14 (13%)	9 (20%)	5 (8%)	0.11
- Thiazide	13 (12%)	8 (18%)	5 (8%)	0.20
- Antimineralocorticoid	6 (6%)	3 (7%)	3 (5%)	0.98
- LABA	18 (17%)	16 (36%)	2 (3%)	<0.001
- LAMA	17 (16%)	17 (38%)	0 (0%)	<0.001
- ICS	5 (5%)	5 (11%)	0 (0%)	0.02
- OCS	0 (0%)	0 (0%)	0 (0%)	
Pulmonary function tests				
- FEV_1_/FVC, %	71 ± 13	58 ± 9	80 ± 5	<0.001
- FEV_1_, % of predicted	80 ± 24	63 ± 18	92 ± 21	<0.001
- TLC, % of predicted	104 ± 20	113 ± 22	97 ± 16	<0.001
- RV, % of predicted	141 ± 47	169 ± 52	121 ± 30	<0.001
- RV/TLC, % of predicted	125 ± 23	138 ± 22	116 ± 19	<0.001
- DLCO/VA, % of predicted	68 ± 19	58 ± 22	71 ± 17	0.06
ABGs				
- SpO_2_, %	94 ± 3	92 ± 3	94 ± 3	0.02
- PaO_2_, mmHg	65 ± 9	63 ± 9	66 ± 10	0.20
- PaCO_2_, mmHg	36 ± 5	37 ± 5	35 ± 5	0.05
- PH	7.4 ± 0.03	7.5 ± 0.02	7.4 ± 0.03	0.10
- Bicarbonate, mmHg	23 ± 2	24 ± 2	23 ± 2	0.81

Values are presented as mean ± standard deviation, median (first; third quartile), or the number of patients (%). ABGs, arterial blood gases; ACE, angiotensin-converting-enzyme; AT_1_, angiotensin II receptor type 1; CCB, calcium channel blocker; CK_max_, maximum creatine kinase; COPD, chronic obstructive pulmonary disease; CRP, C-reactive protein; cTnT_max_, maximum cardiac troponin T; DL_co_/VA, diffusing capacity for carbon monoxide/alveolar volume; FEV_1_, forced expiratory volume in 1 s; FVC, forced vital capacity; GFR, glomerular filtration rate; ICS, inhaled corticosteroid; LABA, long-acting beta-adrenoceptor agonist; LAMA, long-acting muscarinic antagonists; NSTEMI, non-ST elevation myocardial infarction; NT-proBNP, N-terminal pro B-type natriuretic peptide; OCS, oral corticosteroid; PaCO_2_, partial pressure of carbon dioxide; PaO_2_, partial pressure of oxygen; RV, residual volume; SpO_2_, oxygen saturation; STEMI, ST-elevation myocardial infarction; TLC, total lung capacity.

**Table 2:** Echocardiography in patients with versus without COPD.

	COPD (*n* = 45)	Non-COPD (*n* = 64)	*p*-Value
LVEF, %	48 ± 9	53 ± 7	0.01
LVEDD, mm	48 ± 8	49 ± 5	0.40
LVESD, mm	36 ± 10	33 ± 6	0.13
LA_Vol_, mL	61 ± 29	49 ± 17	0.03
LAVI, mL/m^2^	32 ± 14	25 ± 9	0.01
LV-GLS, %	−15 ± 4	−18 ± 4	<0.001
LASr, %	24 ± 10	34 ± 6	<0.001
LAScd, %	−12 ± 6	−19 ± 4	<0.001
LASct, %	−12 ± 5	−15 ± 3	0.002

Values are presented as mean ± standard deviation, median (first; third quartile), or the number of patients (%). LAScd, left atrial strain during conduit phase; LASct, left atrial strain during contraction phase; LASr, left atrial strain during reservoir phase; LAVI, left atrial volume index; LA_VOL_, Left atrial volume; LVEDD, left ventricular end-diastolic diameter; LVEF, left ventricular ejection fraction; LVESD = left ventricular end-systolic diameter; LV-GLS, left ventricular global longitudinal strain.

## Data Availability

Data are available upon reasonable request to the corresponding author.

## Supplementary Materials

The following supporting information can be downloaded at: https://www.mdpi.com/article/10.3390/jcm11071917/s1, Figure S1: Estimated differences in strain values between patients with and without COPD (Strain value of COPD patients—Strain value of non-COPD patients). Positive estimates indicate larger (more positive or less negative) values among COPD patients. Model 1: unadjusted; model 2: adjusted for LVEF (%); model 3: adjusted for LVEF (%) and age (years); model 4: adjusted for TRVmax (m/sec); model 5: adjusted for LVEF (%), age (years) and TRVmax (m/sec). (last variable was available in 73% of COPD echocardiograms and 77% of non-COPD echocardiograms); Figure S2: Post-AMI left ventricular global-longitudinal-strain (LV-GLS) values among patients with and without COPD, comparing patients with and without COPD medication at admission.
