# Supplementary material for: Effects of COPD on Left Ventricular and Left Atrial Deformation in Patients with Acute Myocardial Infarction: Strain Analysis Using Speckle-Tracking Echocardiography
Source: J Clin Med. 2022 Mar 30;11(7):1917. doi: 10.3390/jcm11071917 (PMC8999583; doi:10.3390/jcm11071917)
Supplement: Supplementary file 1 [file jcm-11-01917-s001.zip › jcm-1613562-supplementary.pdf]

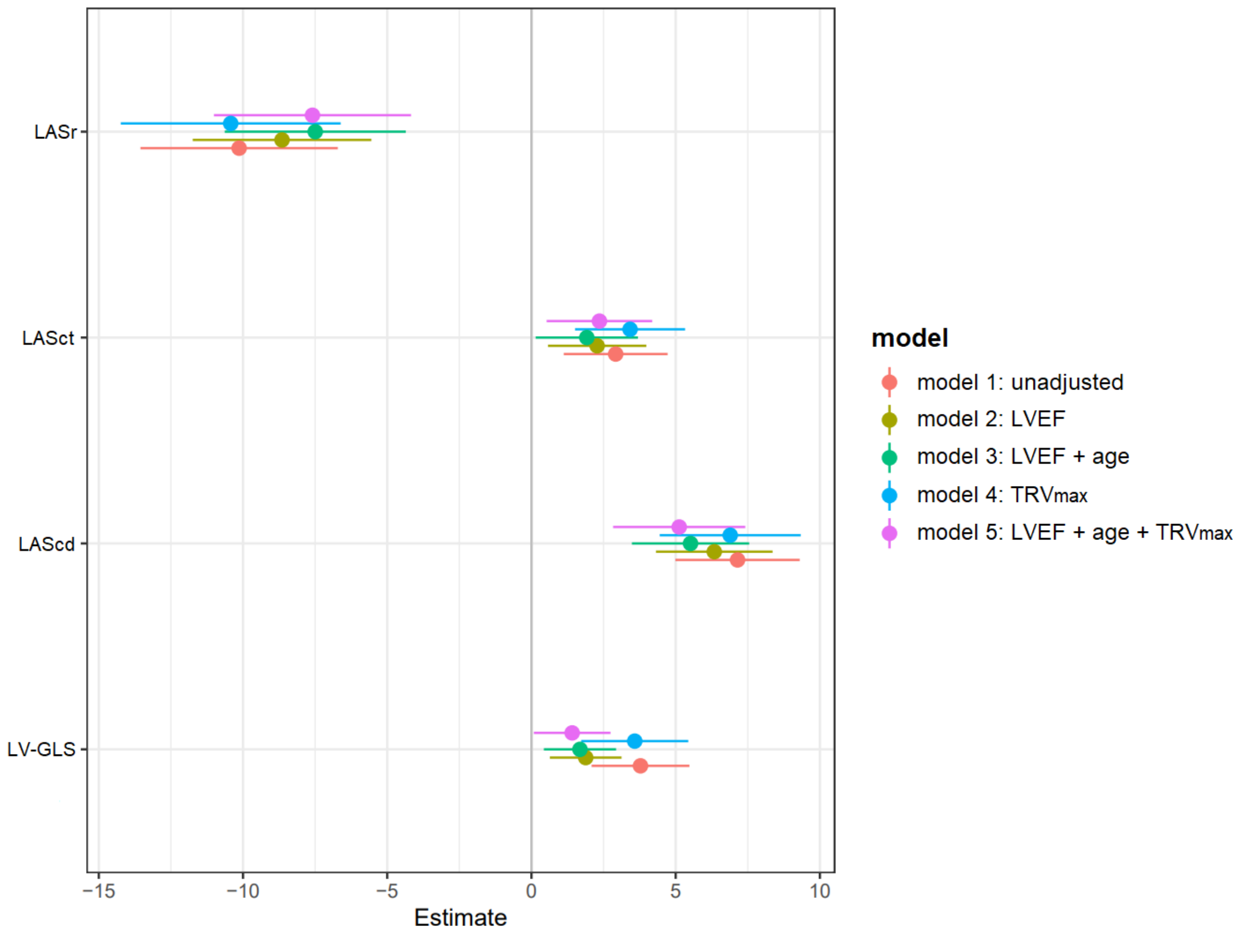

Figure S1. Estimated differences in strain values between patients with and without COPD (Strain value of COPD patients – Strain value of non-COPD patients). Positive estimates indicate larger (more positive or less negative) values among COPD patients. Model 1: unadjusted; model 2: adjusted for LVEF (%); model 3: adjusted for LVEF (%) and age (years); model 4: adjusted for TRVmax (m/sec); model 5: adjusted for LVEF (%), age (years) and TRVmax (m/sec). Abbreviations: COPD, chronic obstructive pulmonary disease; LAScd, left atrial strain during conduit phase; LASct, left atrial strain during con-traction phase; LASr, left atrial strain during reservoir phase; LVEF, left ventricular ejection fraction; LV-GLS, left ventricular global longitudinal strain; TRVmax, maximum tricuspid regurgitation velocity (last variable was available in 73% of COPD echocardiograms and 77% of non-COPD echocardiograms).

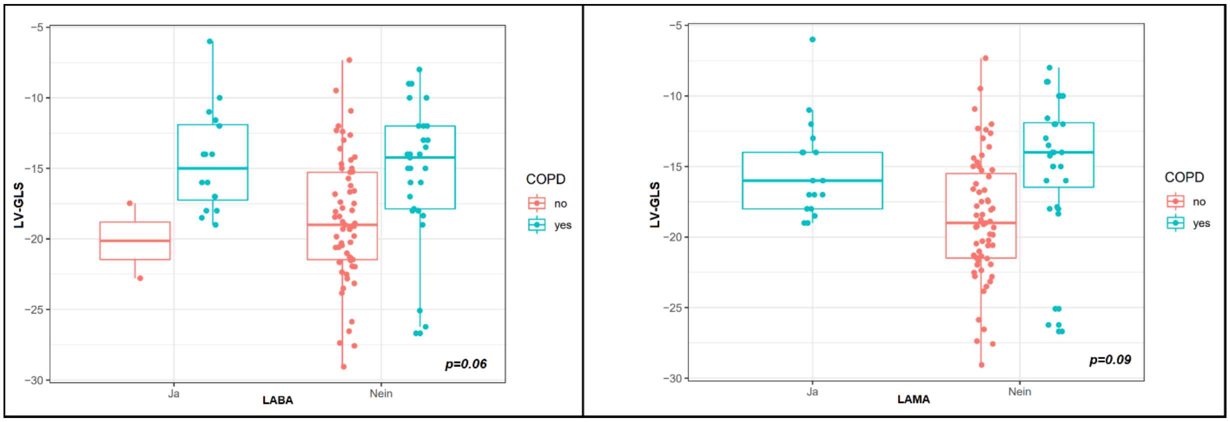

Figure S2. Post-AMI left ventricular global-longitudinal-strain (LV-GLS) values among patients with and without COPD, comparing patients with and without COPD medication at admission. Abbreviations: AMI, acute myocardial infarction; COPD, chronic obstructive pulmonary disease; LABA, Long-acting beta-adrenoceptor agonist; LAMA, Long-acting muscarinic antagonists; LV-GLS, left ventricular global longitudinal strain.
